# Dreading Yet Hoping: Traumatic Loss Impacted by Reference DNA Sample Collection for Families of Missing People

**DOI:** 10.3389/fpsyt.2022.866269

**Published:** 2022-04-04

**Authors:** Sarah Wayland, Jodie Ward

**Affiliations:** ^1^School of Medicine and Health, University of New England, Sydney, NSW, Australia; ^2^National DNA Program for Unidentified and Missing Persons, Australian Federal Police, Canberra, ACT, Australia; ^3^Centre for Forensic Science, University of Technology Sydney, Sydney, NSW, Australia

**Keywords:** missing persons, DNA sample, traumatic loss, unresolved grief, ambiguous loss, unidentified human remains

## Abstract

The trauma of having a family member missing is commonly described as an ambiguous loss where the finality of the loss is not realized, as is experienced with a death. There is uncertainty due to the trauma of the absence and subsequent police investigation, leading to physical and emotional impacts for the aftercare of those left behind. There are 850 unidentified human remains and 2,600 long-term missing persons cases in Australia. The Australian Federal Police (AFP) National DNA Program for Unidentified and Missing Persons aims to scientifically link these cases using modern DNA techniques and databases. A DNA-led identification effort may assist to provide answers to Australian families searching for missing relatives, but may also contribute to the trauma experienced by these families. A literature review demonstrated empirical research for the development of scientific best practices for the collection of reference DNA samples for forensic purposes, but minimal evidence about the impact of reference DNA sample collection on kin when attempting to identify the deceased remains of missing people in non-mass casualty situations. The aim of this study was to develop an academically robust understanding of the unique impact of reference DNA sample collection on families of missing persons and support pathways tailored to the experience. This study involved 26 Australian families of long-term missing (ranging from 1 to 20+ years) people in Australia anonymously completing a mixed-methods online survey about their experiences of providing reference DNA samples to aid missing persons investigations. Respondents were representative of a range of ages, genders and relationships to the missing individual. The thematic analysis of the survey results identified the provision of a reference DNA sample: (1) resembles an overt act of hope as families perceive their sample assists the investigation, whilst also being traumatic, triggered by the prospect of scientifically matching their missing family member to a set of unknown human remains; (2) can cause immediate interpersonal impacts and ongoing impacts to families' wellbeing; and (3) can be improved by considering the environment where the sample is collected, professionalism of the police officer collecting the sample, timeliness of the provision of the sample, level of support provided during and after sample collection, and effective communication of forensic procedures and processes as they relate to the missing persons investigation. The study concludes that the complexity associated with provision of family reference samples requires the development and implementation of best practice guidelines, including psycho-education strategies to be used by practitioners to minimize the vicarious trauma for relatives already traumatized by the loss of their missing family member. These guidelines would support the objectives of the AFP Program and benefit all routine missing persons investigations.

## Introduction

In Australia, a missing person is defined as a person whose whereabouts are unknown and there are concerns for their safety and wellbeing. Recent research from the Australian Institute of Criminology (1) note that people go missing for various reasons, including complex mental health conditions, concerns regarding suicidality, they are victims of crime or they are young people seeking independence. Every year in Australia, more than 40,000 reports are made in relation to a missing person (1). Those who are reported missing are likely to return within 1 month, with long-term missing people (defined as missing for longer than 3 months) accounting for 2,600 Australians at present. A nation-wide audit led by the Australian Federal Police (AFP) National DNA Program for Unidentified and Missing Persons (AFP Program) has recently recorded 850 unidentified humans remain in Australia; a higher figure than initial estimates of 500 (2, 3). The expectation is that some of these unknown remains will be linked to known missing persons, who in some cases have been absent for decades (3). To achieve this, the AFP Program will use modern DNA techniques and databases to match the DNA from unknown deceased persons to DNA volunteered by missing person relatives on a national level for the first time in Australia.

When unidentified human remains are recovered, the process of DNA profiling and matching has been shown to provide scientific support regarding the unknown deceased person's identity following large-scale disasters (4, 5). A review of the literature identified that there is empirical research regarding the optimal process of reference DNA sample collection for forensic purposes, including recommendations for the specific type and number of reference DNA samples that should be collected from biological relatives of a missing person or disaster victim (3, 6). Additionally, there are recently published international guidelines for police and forensic investigators regarding the use of DNA for humanitarian and mass disaster operations (7–9), and publicly available information brochures [e.g., (10)] and online resources (e.g., https://www.missingpersons.gov.au/support/national-dna-program-unidentified-and-missing-persons) for families to aid their understanding of the use of DNA for identifying human remains. However, there is minimal evidence, and therefore consideration, of the impact of reference DNA sample collection on kin when attempting to identify the remains of unknown deceased persons for domestic missing persons cases (i.e., non-mass casualty events).

The trauma of having a family member missing is commonly described as an ambiguous loss (11, 12), where the finality of the loss is not realized and families exist in the liminal space between the person being “both here and gone” (13, 14). The care required after the loss is different to a death where there is uncertainty for those left behind where they can be physically and emotionally impacted (15). This liminality is identified by time periods where ideas of uncertainty are “played out” by family members where the potential for return is viewed alongside ideas of never returning (16). The experience of being “left behind” when someone is missing is viewed as a traumatic event characterized by reactions of waiting and loss of control, irrespective of the length of time the person is missing (17). Research identifies that the longer-term impact of having someone missing does not present like grief and loss reactions, where the loss is able to be managed over time (18). Ambiguous loss, where the person remains missing, can exist for decades given families do not have the necessary information to be able to state with certainty that a person will or will not come home, in addition to thoughts about being alive or deceased (14).

Parker and colleagues (19), reflecting on the ethical impacts of using DNA to identify human remains, note that DNA identification has both social and individual goals. The humanitarian goal to confirm who a person is, offers families awaiting news about the traumatic loss of a missing person the chance to perform funerary rights. This in turn provides psychosocial benefits to families waiting for information. Parker and colleagues (19) also highlight that the psychological impact of a traumatic event, such as having a person you love go missing, can be compounded by the social isolation that occurs due to lack of certainty, resulting in psychological pain. Identification of an individual can address feelings of exclusion because they offer certainty that, socially, is likely to be accepted by the community. The disenfranchised grief, as noted by Doka (20), of having someone missing can be resolved by certainty as to their whereabouts with an ambiguous or “non death related” (pg. 7) loss being non-linear, creating no clear trajectory as to how people may move away from their loss given its unresolved nature. In addition to Doka's work (20), Boss (13, 14) notes that families left behind can be frozen in time, in response to their grief, where the situation is locked to the time the person vanished or to the lack of information required to accept the loss.

Research in this space is focused on the experiences of families with family members unaccounted for following mass casualty events. As an example, the human rights organization “Abuelas de Plaza de Mayo”, which was formed by the grandmothers of the Plaza de Mayo in response to the many families still searching for loved ones missing since the last Argentinian dictatorship from 1976 to 1983 [see (21)], have published real-time observations of continuing to search for the missing and the impact of forensic processes. The remainder of the literature also relates to identity formation in the sense of people being able to understand who they are and where they belong, as a result of the use of reference DNA samples, for those disconnected from family due to armed conflict, political unrest, or natural and man-made mass disasters (22, 23). This literature provides context to awareness regarding the impact of searching for previous generations who are presumed deceased (24), but not to present day searching for long-term missing people.

Provision of a reference DNA sample may be an overt act of hope for some families by being able to provide information to help the investigation, or it may be a traumatic event that signifies that identification of human remains cannot be conducted visually, and requires the undertaking of scientific matching processes due to the condition of the remains and/or the procedural requirements of the jurisdiction. In 2015, the INTERPOL DNA monitoring expert group [see (9)] noted that there was a need for both national and international cooperation in missing persons investigations because “families have a fundamental right to know, and the right to justice” (pg. 2). Unlike DNA identification of disaster victims, which are typically identified rapidly due to the high profile and public nature of the event, community expectations and provision of adequate resources, DNA identification may take an extended period of time for routine missing persons cases (25). This has previously been reported to be the case in Australia, despite advances in DNA technology and databases over the last decade. This paper reflects on ways to ensure that the lived experience of families of missing people can be considered as part of the development of national best practices for missing persons investigations being undertaken as an objective of the AFP Program.

The impact of provision of a reference DNA sample, from the perspective of those providing the sample, has not yet been researched despite the practice becoming common when working with families of long-term missing people, as noted in standard operating procedures prepared for law enforcement and related agencies [e.g., The SOS Guide, National Missing Persons Coordination Centre, n.d., see (26)]. The aim of this qualitative study was to investigate how, as potentially traumatized individuals, families of missing people negotiate the process of providing a reference DNA sample by reflecting on the impact of that sample on their loss journey. We hypothesized that there is minimal awareness of the emotional toll on those relatives providing a family reference sample during a missing persons investigation, with the focus being on the procedural requirements. The aim of this study was to provide a platform for the perspectives of those with lived experience to be considered in the development of best practice family reference sample collection guidelines, given their current absence from the academic literature and policy making processes.

## Materials and Methods

### Respondents

The sample was composed of 26 consenting adults (aged over 18 years) who self-identified as family members of a long-term missing person based in Australia (noting that the person could be missing in Australia or in another jurisdiction). Participants were asked to complete an anonymous survey shared as a live URL or QR code from June to November 2021.

The online survey was circulated via social media through a dedicated research page on Facebook, in addition to the social media pages of peak Australian organizations working with families of missing people to reduce selection bias. The Facebook page provided anonymity and ethical assurances to family members who wished to participate; either regarding safety of their responses in terms of how they are treated by the research team or for those who wanted to engage externally to their connection with Police. People who may have criminogenic histories, have an insecure relationship with police due to historical trauma or political interventions, as well as those that are ethically opposed to provision of a DNA sample, were able to share their perspectives to shape the evidence base (27). The use of an online survey allowed respondents to complete the survey at a time and in a place of their choosing. Respondents were assured that their reference DNA sample would not be linked to their survey responses given the anonymous nature of the data collection and that the processing of their reference DNA sample was independent to their decision to participate in some or all components of this study. Given the study was heavily reliant on qualitative data, collection matters of statistical or power issues were not relevant.

### Data Collection and Data Analysis

The study utilized a mixed-methods online survey (see Appendix A) involving basic demographic characteristics of the respondent and open-ended questions using a descriptive analysis technique (28). Using both quantitative (closed-ended) and qualitative (open-ended) data, and then combining the two, the research team was able to answer their enquiry about the impact of reference DNA sample collection on families of missing people.

The analysis was informed by Braun and Clarke's (29) exploration of reflective thematic analysis, that the lead researcher is proficient in using across missing persons and suicide prevention projects. Saturation of themes was used to determine if there was adequate data to ensure the research question could be answered, and if the responses provided rich narratives to better understand the participant's lived experiences. The focus was to explore the learnt experience, coupled with the shared lived experience of being the family member of a missing person, to better understand how their stories are told and how they are perceived to be interpreted. The survey also sought to identify how families can be better supported in the future, to inform the development of best practices for the collection of family reference samples. Consensus of themes was achieved via recursive practice between the research team to ensure accuracy (30). The goal was to develop an academically robust understanding of the unique impact the collection of a reference DNA sample has on family members when a loved one is missing and to understand support pathways that are tailored to the experience.

Survey transcripts were analyzed independently by two coders using both an inductive and deductive approach similar to that described in Azungah (31). Preliminary analysis of each individual response was conducted as the data were collected. An audit trail of the analytic logic employed by the research team was maintained throughout data collection and analysis (32).

Data from each response were deductively coded against the study aims and data outlying to the aims were inductively coded to ensure that other experiences shared by respondents were not lost. Minor categories within each response were identified. During the second layer of coding, related or contrasting categories across responses were grouped. Outlying categories were not forced to fit into these groupings nor removed from analysis until their relevance was determined at a later stage. A third layer of coding identified minor themes across the categories, which was followed by the grouping of these into major themes. All categories and themes were tested among the research team at all stages of the analytic process for confirmability and dependability (32).

## Results

### Respondent Characteristics

Anonymous survey data identified basic demographics (e.g., age, gender, location, status of missing persons case, cultural background and awareness of DNA procedures), followed by responses to short answer questions regarding their lived experience regarding decision making, experience of provision of a reference DNA sample, and emotional response to the sampling process.

Of the 26 respondents, more than half (15 respondents) represented parents or siblings of the missing person, and the timeframe for relatives being missing ranged from 1 to 5 years (12 respondents) to more than 20 years in some cases (3 respondents). The respondents' self-declared ages spanned from between 20 and 30 years (2 respondents) to over 70 (5 respondents), with a higher representation of respondents identifying as female (18 respondents). In terms of their knowledge about how DNA can assist a missing persons investigation, more than half of the respondents felt somewhat (8 respondents) or completely (7 respondents) confident about their understanding of the process.

### Thematic Analysis

From the analysis of the qualitative data provided by the 26 respondents, three major themes were identified inductively. Respondent demographics are not recorded next to quotes to ensure privacy and minimize reidentification, however the relationship to the absent person and length of time missing are noted.

#### Theme 1: Uncovering Potential Trauma Associated With Provision of a Reference DNA Sample

The identification of the liminal space between the acceptance that a loved one was missing and concern for the potential finality of the unidentified human remains being confirmed as their missing person was revealed in some of the long text responses provided:

“*It's sobering to think of my loved one being deceased and in a state of decomposition.”* (Sibling of a person missing for more than 10 years.)

“*DNA (matching) means there is no hope he is alive. I carry both hope and fear*.” (Sibling of a person missing for more than 5 years.)

“*It's sad that this is required to identify him.”* (Mother of a person missing for between 1 and 5 years.)

“*I was both dreading and hoping that it would lead to a match, as well as hoping for peace of mind that it meant he couldn't/wouldn't be one of those cases we've all heard about where remains are left unidentified for years and years.”* (Sibling of a person missing for more than 5 years; located deceased.)

Within this thematic analysis, the respondents also noted that the provision of the reference DNA sample may suggest the implication, to others, that their missing relative is deceased; an external sign of diminished hope created guilt by suggesting they were, as described by one respondent as “*not hopeful”:*

“*I want the match to be made AND I hope the match won't be made.”* (Sibling of a person missing for <1 year.)

This theme also identified a sub-theme focusing on what was described as “*the active step”* of providing a reference DNA sample. This active, rather than passive, step offered self-described power, in a traditionally disempowered space that occurs when waiting for news. Families referred to providing a reference DNA sample, even without information as to how long a resolution may take, as allowing them to “*seek (potential) closure”*, noting that:

“*DNA could be the key one day, to finally bring him home.”* (Mother of a person missing for between 1 and 5 years.)

“*The notion that this task of providing DNA might get us out of our stuckness was important and empowering. It was something I could actually do after much of the 'doing' – namely searching – had been exhausted).”* (Sibling of a person missing for 5 years and then located deceased.)

“*It made it very real. It made us think about and be open to the idea the he could be deceased which is really hard because you are always hoping that they are still alive. It also provided hope and the feeling that we were doing something proactive.”* (Sibling of a person missing for 7 years and then located deceased 4 years ago.)

Respondents were also capable, in the midst of exposure to potential trauma, to separate the action and focus on the scientific advancements; “*I'm happy to see technology developing”* explained the father of a son missing for more than 10 years. Similarly, a mother with a son missing for more than 5 years reflected “*at least if it's a body/skeleton/unknown person behind the scenes (it means) I have a glimmer of hope to find my son”*.

Providing a reference DNA sample also provided a forward motion for families, as noted by a father of a missing person absent for 20 years; “*I looked at the process as a way of moving forward in an attempt to find out what happened to our son”*. This was similar to other respondents who noted that the active, rather than passive, waiting for news offered by the capacity to provide a reference DNA sample for comparison; specifically, “*it made me feel like something was happening; like having it done might get us one step closer to resolution”*.

For some respondents the act of providing a reference DNA sample was viewed through a procedural lens, where ideas about the emotional impact were held over, for the future:

“*The only emotional aspect that has regularly come to mind is the future turmoil every time human remains are found - that always has and always will be the hardest part of this process for me.”* (Mother of a person missing for 10 years.)

For others, there was the intertwining as to what a DNA match might mean in terms of what happened to their loved one:

“*You start to think of different possible scenarios and your conflicting hope that you do find them but not by connecting DNA samples because that means they have passed. You want to know what's happened but you don't want that to be the outcome.”* (Father of a person missing for more than 10 years.)

#### Theme 2: Practicalities and Procedures When Providing a Reference DNA Sample

Survey respondents reflected that the emotional impact of the process of providing a reference DNA sample was undeniable, as evidenced by the qualitative exemplar quotes from theme one. The respondents suggested that their engagement and interactions with police were at times positive, despite the reports of traumatic loss. In the survey, respondents were asked to rate their experience of the reference DNA sample collection process using a scale (positive, neutral or negative), prior to completing their free text responses. Only one respondent recorded a negative experience, with the remainder noting either a positive or neutral experience, despite the richness of the narratives about the traumatic impact.

From a procedural perspective, the recording of positive or neutral responses reflected the families' belief that police made them feel safe and supported during the reference DNA sample collection process. Narrative responses referred to individual behaviors of police, who demonstrated compassionate responses and gratitude for the family's contribution to the missing persons investigation. However, these reflections were outweighed by those family members who felt their reference DNA sample provision required better management. The sub-themes of the qualitative responses identified issues relating to the environment where the sample was collected, professionalism of the police officer collecting the sample, timeliness of the provision of the sample, level of support provided during and after sample collection, and knowledge and communication of forensic procedures and processes as they relate to the missing persons investigation.

#### Environment

Respondents noted that samples were taken in their home, in their workplace or at a police station; with many families finding attending police stations or police attending workplaces stressful or fostered a clinical experience.

“*The environment was sterile, and unfamiliar (police headquarters), without any space to acknowledge the gravity of the situation. It was very transactional.”* (Sibling of a person missing for 5 years and then located deceased.)

Another family member (parent of a person missing for more than 20 years) provided a sample in their workplace and felt this was “*insensitive”*, with the office*r “treating it as if it was routine”*.

#### Police Professionalism

Respondents reflected on the ways in which they encountered the police officer tasked with collecting the sample, and the way this impacted their distress:

“*(Families) have already run all the possible scenarios through their heads 1000 times over but not let themselves believe the worst. So, they need sensitivity and understanding because this is a real person and this is real life. No one could ever understand what it feels like unless they have been through it*.” (Sibling of a person missing for more than 20 years.)

Respondents identified that it appeared as though some police officers had limited understanding of DNA identification procedures or these were unable to be effectively communicated to all family members, which enhanced feelings of uncertainty and left families unsupported. For example, “*one police officer has no true understanding of the process for missing persons, and how families might feel”* explained the mother of a missing child, absent for more than 5 years.

The ability of the police officers collecting the sample to satisfactorily answer questions about the storage of the sample, the legalities of what their DNA could be used for and the scientific approach used for human remains identification was an important step to make families feel emotionally safe during the process.

#### Timing

Long-term missing persons investigations, as identified by the respondents, were subject to changing procedures from police, during the months, years or decades since a disappearance. Respondents noted that the request for family members to provide a reference DNA sample had been conducted in an ad-hoc way in the past. This was often dependent on the purported circumstances as to why or how a person vanished (i.e., identifying if the absence was self-motivated or related to being a victim of a crime) or how long the person had been absent for.

Respondents noted that decisions regarding a request to kin for a reference DNA sample was either made via the investigating police officers, a Coronial enquiry into an absence or families proactively approached police as a way to ensure that every action had been undertaken to assist with seeking finality. The sibling of a person missing for 5 years noted:

“*I can't stress enough how important I feel it is that samples are collected in the early stages of a disappearance, not solely from an administrative perspective, but so that loved ones aren't providing samples after the fact (as per what happened with my parent after his remains were found). It is far more traumatising a context to be giving a sample in, and completely avoidable additional trauma.”*

Families also noted that finding out about the recovery of human remains from media broadcasts, rather than being contacted to provide a reference DNA sample based on the possibility that it may be their loved one, was “*emotionally confusing, it added another layer of distress*”. Learning from a secondhand source like the media, rather than the investigating police, was described by the father of a missing son as “*demoralising to read of such issues being reported in the press and in media without being advised beforehand”*.

#### Forensic Procedures and Processes

Procedurally, families reflected on some ambiguity in the process described or demonstrated to them about their reference DNA sample:

“*A clear outline of what the DNA (sample) could be used for was left deliberately ambiguous by (name of police jurisdiction*).” (Sibling of a missing person, since located deceased.)

For families who had a loved one missing outside of their home country, concerns regarding the safety of their sample were noted, as well as concerns that the police officer taking the sample might not be privy to the next steps of the forensic process.

“*I felt it was in incompetent hands”* explained the mother of a missing child, when (what the participant described as a) *junior* police officer was sent to collect the sample. Respondents noted that family members required certainty that samples would be collected both in a timely and competent manner, and with compassion.

#### Theme 3: Constructing Recommendations for Future Engagement Between Families and Practitioners Collecting Reference DNA Samples

In the final questions of the qualitative survey, respondents were asked to consider the ways in which reference DNA sample collection may be enhanced to support their journey of ambiguous loss. The underlying themes, deductively identified, suggested that the intersection between practical and emotional recognition needed to be underpinned by “*honesty and openness”* from policing jurisdictions:

“*We wanted a testament from the officer about the importance (of what we were providing) and some gratitude of the contribution we were making to the missing persons investigation.”* (Sibling of a person missing for 5 years.)

Recommendations for enhanced privacy relating to the location of where the sample would be collected, the handling and storage of the sample, and the handling and storage of the resulting DNA profile were identified by the respondents. For example, “*I want them to be sensitive (to family's needs), I have an expectation of the need for an outline as to where the sample would go and who I would follow up with”* explained the mother of a missing person.

Some respondents noted that support was required during and after the provision of a sample. It was suggested by some respondents that police officers could attend homes without uniforms to soften the experience and provide information about referral services for families wishing to seek such services after providing a sample. As explained by the sibling of a missing person, missing for 5 years:

“(what we need for collection is a…) *A neutral space. Police not wearing uniforms (perhaps just badges) ….and the process in detail including what will happen next – where it goes, how it will be stored/checked against, and how long it will take for the DNA sample to land where it needs to (weeks? months?). Then of course where they can go if they'd like more assistance before/after.”*

## Discussion

The respondents to this Australian survey of families of long-term missing people identified that the provision of a reference DNA sample was a potential trauma attached to their unresolved loss, through pre-occupation about human remains, or confirmation of the finality of their loss.

The survey analysis identified that much of the lived experience of having someone missing, involves real and imagined traumas about where the missing person may be, what has happened to them and when the loss will be resolved. Missing persons are people at risk, with this risk extending from the reasons why they are absent, to their welfare whilst their whereabouts are unknown (27). There are layers or elements of trauma that can be continually disempowering and confusing in terms of the realities of living with a traumatic loss that has no end, consistent with the evidence base exploring complicated grief, bereavement and ambiguous loss (14).

What was identified with the families who took part in this study, as distinctly different to the literature that focuses on victim identification following a mass disaster, is that the collection of a reference DNA sample can exacerbate grief ruminations (17) for the sample donor in situations where only one loss has occurred. These grief ruminations can offer insight into the impacts when only one loss occurs, compared to when there are multiple fatalities. These grief ruminations can include imagined aspects of the person's physical degradation, or even the likelihood that remains still exist. Without the connection to a group of others, such as in mass casualty events, who have experienced similar losses at the same time, families of individual missing experiences may be unaware that others are also living through similar experiences (9). The respondents to the survey noted that elements of the liminal space between hopefulness and hopelessness exist, even when practical aspects such as providing a reference DNA sample occur. For some families, the act of providing a sample may signify that they have “*given up hope”* for the safe return of the missing person, or that offering the sample suggests that their idea of a safe return may no longer be possible. Earlier work by author one noted that this concept of adding “*but.maybe”* to lived experience reflections of having someone missing meant that disappearances were characterized by a lack of extinguishment of hope for a possible return. This shifting to a potential reunion with physical remains is both traumatic for individuals to conceptualize and comforting in terms of finality (16).

Likewise, much of the previous literature on the lived experience of families of missing people refers to closure as not being possible for families of missing people given the inherent ambiguity of cases. Melnick and Roos (33) argue that closure has been a common feature of the social discourse surrounding response to loss where, however in the case of ambiguous loss, adjustment to life post-loss is not dependent on completing elements of grief reactions where closure may typically inhabit. When a disappearance occurs, each piece of information about the whereabouts of a person who is lost adds to iterative moments that lead to potential answers. However, in this study families spoke of the reference DNA sample providing a segue to potential closure, where the concept of closure was welcomed. The scientific certainty of their reference DNA sample “*matching”* to unidentified human remains offers finality in a way that no other information can provide; even in cases where a coronial investigation declares a person is deceased but no body has yet been located (34). Therefore, may alleviate the depression and prolonged grief disorder in family members of missing individuals, as seen in previous mass casualty events such as the 2004 tsunami, however it is unclear if these findings can be applied in single missing persons events due to the multifactorial reasons as to how and why people go missing, and what other impacts there may be on their mental health (35). Families may welcome the opportunity to provide a reference DNA sample to achieve this outcome, therefore challenging previous research (17) and requires further exploration in terms of developing strategies to assist families of the missing to live with their traumatic losses.

The additional finding of the study situated the role of “*others”*, and their significant impact on the wellbeing of those living with a loss. The role of police officers and their interpersonal skills in conveying empathy, compassion and clarity regarding the forensic procedure had a direct impact on the wellbeing of families during and after the provision of a sample. This is reflective of the literature on the certainty of those in positions of power offering warmth and connection to those experiencing trauma—particularly in the forensic field where sensitive engagement with families relating to human remains is noted (14). Enhanced training of police in both the scientific sampling of DNA and the ways in which this is verbally relayed to families may minimize vicarious trauma of both the police officer providing assistance and the family whose sample has been taken (23).

Finally, the literature reviewed in the earlier stages of this study identified that there are national and international guidelines relating to disaster victim identification—these map the connections between engagement with the scientific process and families' experiences of loss, in addition to awareness of their psychosocial needs (22). However, the desperate nature of single missing persons investigations where there is one person and one family are not covered by the same guidelines, despite the families having similar presentations to those involved in large-scale events like natural or man-made disasters. The International Committee of the Red Cross (ICRC) proposed a best practice guide for the DNA identification of missing and unidentified persons in situations of armed conflict or violence (23) that includes ethical and legal issues, but no context regarding emotional impacts. The resource did note the sensitive nature of a person's DNA and the concerns regarding the appropriate storage, handling and use of the DNA information, however the psychological or emotional impacts of the process of providing a sample, or the associated trauma of connecting the sample to a set of human remains, is not included.

The Australian Law Reform Commission's (2010) “*Essentially yours: The protection of human genetic information in Australia”* (36) report provides procedural, legal and privacy regulations regarding the collection and use of DNA samples that are voluntarily provided by kin. The only gray literature that links emotional support with the procedural awareness as to how DNA and forensic collection may impact families is in the AFP guide “*The SOS Guide: A guide for the families of missing people”* (26); with a section identifying emotional impacts. There are minimal resources that explore the process and traumatic impact, outside of scientific approaches or consent guidelines for families [see (37, 38)]. Ensuring that all practitioners collecting samples should complete ambiguous loss training as part of their core training requirements, to better support the aftercare needs of families of missing people, may assist in managing exposure to repetitive trauma.

Awareness of the connections between science, consent and emotional support needs may enhance the education requirements for professionals involved in single missing persons cases. Furthermore, the psycho-education needs of missing persons relatives should be treated through the lens of both the trauma response, and the procedural engagement. These concepts are worthy of future investigation.

## Limitations

As with all research methods there are limitations due to the research design, when utilizing survey to elicit responses. The capacity to reach out to participants, during a time of significant covid impacts in Australia, provided scope for individuals to connect with the research in their own homes. Potentially enhancing the response rate. Additionally, the qualitative focus of the survey limits capacity to clarify or confirm responses with participants, however the depth of responses from respondents provided scope to understand the impact of a DNA sample and the narratives of their lived experiences in relation to an ambiguous loss. Considering the sample size, the research team were able to reach saturation of themes confirming research rigor and confidence in the research findings.

## Conclusion

This study further extends the awareness that ambiguous loss and grief caused by the disappearance of a loved one can be considered by both the impact of the emotional and the practical requirements, and in this sense comparable to the effects of ambiguous loss. These presentations, in response to reference DNA sample collection, are linked to the experiences of ambiguous loss, where the lack of certainty about the finality of the loss and the waiting for confirmation of identity of human remains can provide cumulative impacts in an already traumatic situation. In this context, our respondents described the ways in which their personal reflections on the gravity of the provision of a sample, coupled with the professional behavior of police collecting the sample, offered an overt act of hope to enable them to consider the finality of their loss. The development and implementation of best practice guidelines for family reference sample collection considerate of both the technical and emotional aspects of undergoing a forensic procedure, outside those prepared for mass disasters will assist to minimize the vicarious trauma experienced by relatives already traumatized by the loss of their missing family member. Additionally, these guidelines should contribute to improving the families' awareness that there is a commonality of experience despite their lack of connection with other families in similar situations.

## Data Availability Statement

The raw data supporting the conclusions of this article will be made available by the authors, without undue reservation.

## Ethics Statement

The studies involving human participants were reviewed and approved by University of New England Human Research Ethics Committee (HE21-136). The patients/participants provided their written informed consent to participate in this study.

## Author Contributions

SW is the lead researcher on the project. Both SW and JW designed the study, with SW overseeing the recruitment strategy and data collection period. Both researchers were involved in data analysis, with SW leading the writing of the manuscript and JW assisting with editing the manuscript and the scientific components of the literature review to ensure accuracy. Both authors approved the submitted version.

## Conflict of Interest

The authors declare that the research was conducted in the absence of any commercial or financial relationships that could be construed as a potential conflict of interest.

## Publisher's Note

All claims expressed in this article are solely those of the authors and do not necessarily represent those of their affiliated organizations, or those of the publisher, the editors and the reviewers. Any product that may be evaluated in this article, or claim that may be made by its manufacturer, is not guaranteed or endorsed by the publisher.
