# Corrigendum: Dreading Yet Hoping: Traumatic Loss Impacted by Reference DNA Sample Collection for Families of Missing People

**DOI:** 10.3389/fpsyt.2022.940169

**Published:** 2022-08-19

**Authors:** Sarah Wayland, Jodie Ward

**Affiliations:** ^1^School of Medicine and Health, University of New England, Sydney, NSW, Australia; ^2^National DNA Program for Unidentified and Missing Persons, Australian Federal Police, Canberra, ACT, Australia; ^3^Centre for Forensic Science, University of Technology Sydney, Sydney, NSW, Australia

**Keywords:** missing persons, DNA sample, traumatic loss, unresolved grief, ambiguous loss, unidentified human remains

In the original article, the reference for (9) “Grewcock M. Slipping through the net? Some thoughts on the Cornelia Rau and Vivian Alvarez inquiry. *Curr Issues Crim Just*. (2005) 17:284–90. doi: 10.1080/10345329.2005.12036355” was included but not referenced. It has been removed from the reference list.

In the original article, the reference for (26) “Ward J. Best practice recommendations for the establishment of a national DNA identification program for missing persons: a global perspective. *Forensic Sci Int: Genet Suppl Ser*. (2017) 6:e43-e45. doi: 10.1016/j.fsigss.2017.09.009” was included but not referenced. It has been removed from the reference list.

In the original article, the reference for (36) “Isuru A, Hewage SN, Bandumithra P, Williams SS. Unconfirmed death as a predictor of psychological morbidity in family members of disappeared persons. *Psychol Med*. (2019) 49:2764–71. doi: 10.1017/S0033291718003793” was included but not referenced. It has been removed from the reference list.

In the original article, reference (3), “Ward J. The past, present and future state of missing persons investigations in Australia. *Aus J For Sci*. (2018) 50:708–22. doi: 10.1080/00450618.2018.1466535” was not cited in the article. The citation has now been inserted in Section **Introduction**, Paragraph one:

“The expectation is that some of these unknown remains will be linked to known missing persons, who in some cases have been absent for decades (3).”

In the original article, reference (10), “President's DNA Initiative. *Identifying Victims Using DNA: A Guide for Families*. Washington, DC: U.S. Department of Justice (2005). Available online at: https://www.ojp.gov/ncjrs/virtual-library/abstracts/identifying-victims-using-dna-guide-families-guia-para-las-familias” was not cited in the article. The citation has now been inserted in Section **Introduction**, paragraph two:

“Additionally, there are recently published international guidelines for police and forensic investigators regarding the use of DNA for humanitarian and mass disaster operations (7–9), and publicly available information brochures [e.g., (10)] and online resources (e.g., https://www.missingpersons.gov.au/support/national-dna-program-unidentified-and-missing-persons) for families to aid their understanding of the use of DNA for identifying human remains.”

In the original article, reference (25), “Gin K, Tovar J, Bartelink EJ, Kendell A, Milligan C, Willey P, et al. The 2018 California wildfires: integration of rapid DNA to dramatically accelerate victim identification. *J For Sci*. (2020) 65:791–9. doi: 10.1111/1556-4029.14284” was not cited in the article. The citation has now been inserted in **Introduction**, Paragraph six:

“Unlike DNA identification of disaster victims, which are typically identified rapidly due to the high profile and public nature of the event, community expectations and provision of adequate resources, DNA identification may take an extended period of time for routine missing persons cases (25).”

The references have been renumbered as a result of other reference updates.

The authors apologize for this error and state that this does not change the scientific conclusions of the article in any way. The updated reference list appears below. The original article has been updated.

## Publisher's Note

All claims expressed in this article are solely those of the authors and do not necessarily represent those of their affiliated organizations, or those of the publisher, the editors and the reviewers. Any product that may be evaluated in this article, or claim that may be made by its manufacturer, is not guaranteed or endorsed by the publisher.
