# Supplementary material for: Dreading Yet Hoping: Traumatic Loss Impacted by Reference DNA Sample Collection for Families of Missing People
Source: Front Psychiatry. 2022 Apr 4;13:866269. doi: 10.3389/fpsyt.2022.866269 (PMC9013849; doi:10.3389/fpsyt.2022.866269)

Supplementary Table 1

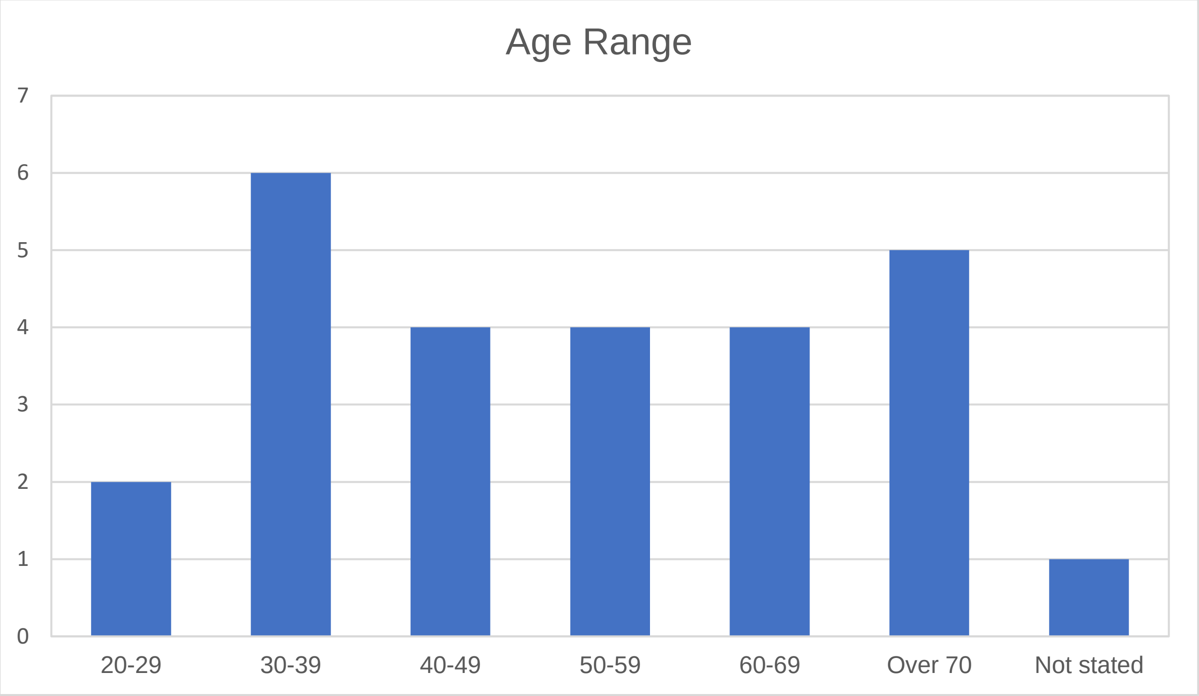

Supplementary Table 2

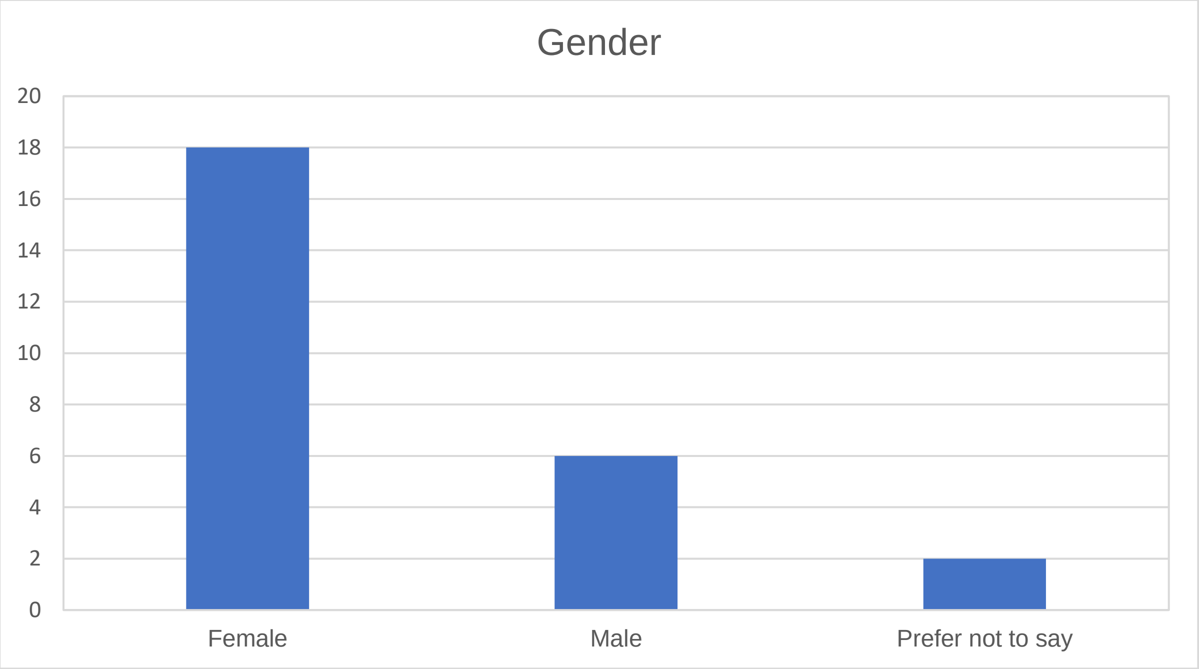

Supplementary Table 3

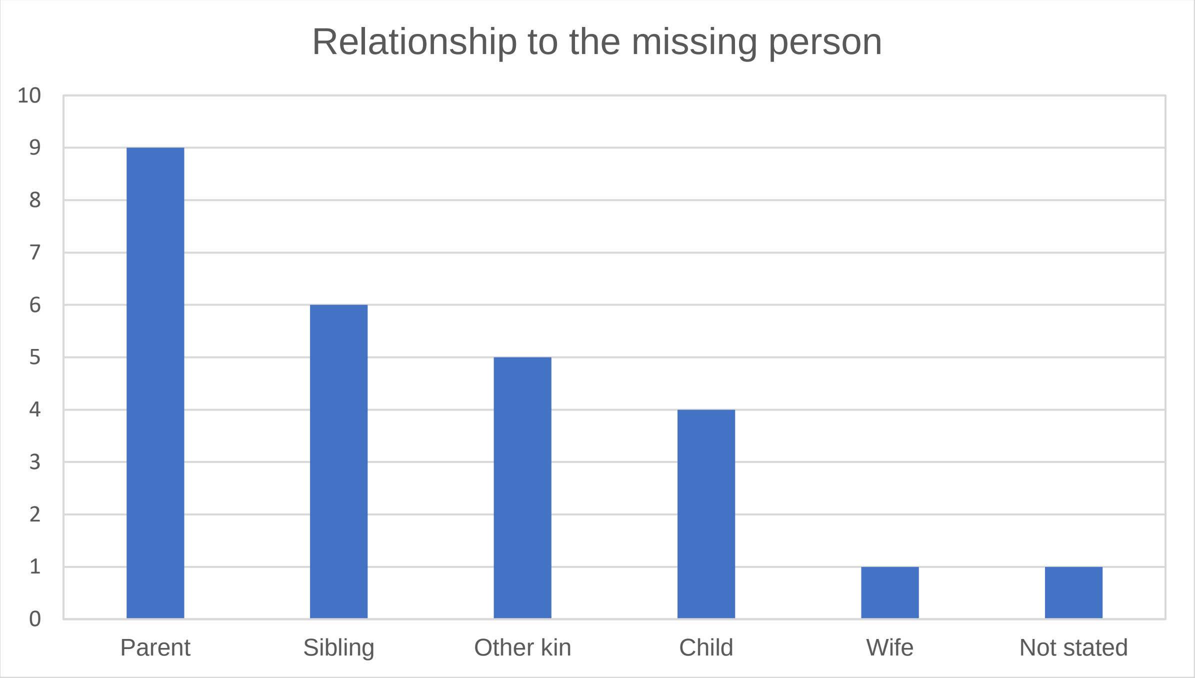

Supplementary Table 4

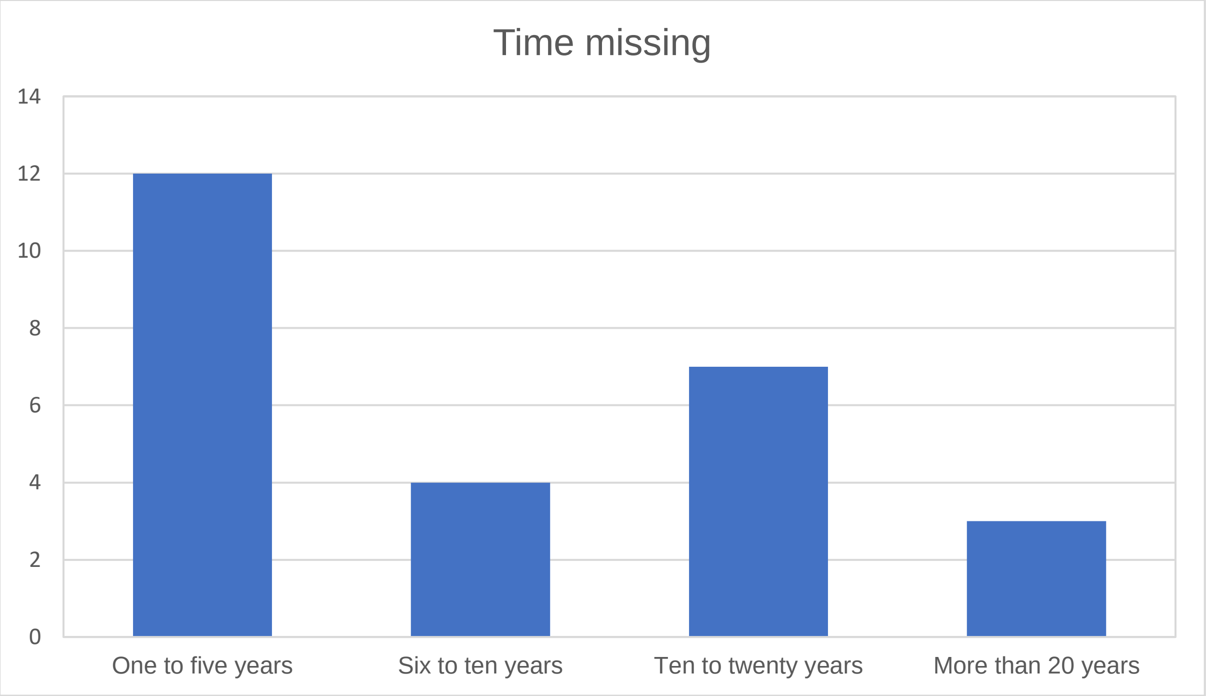

Supplementary Table 5

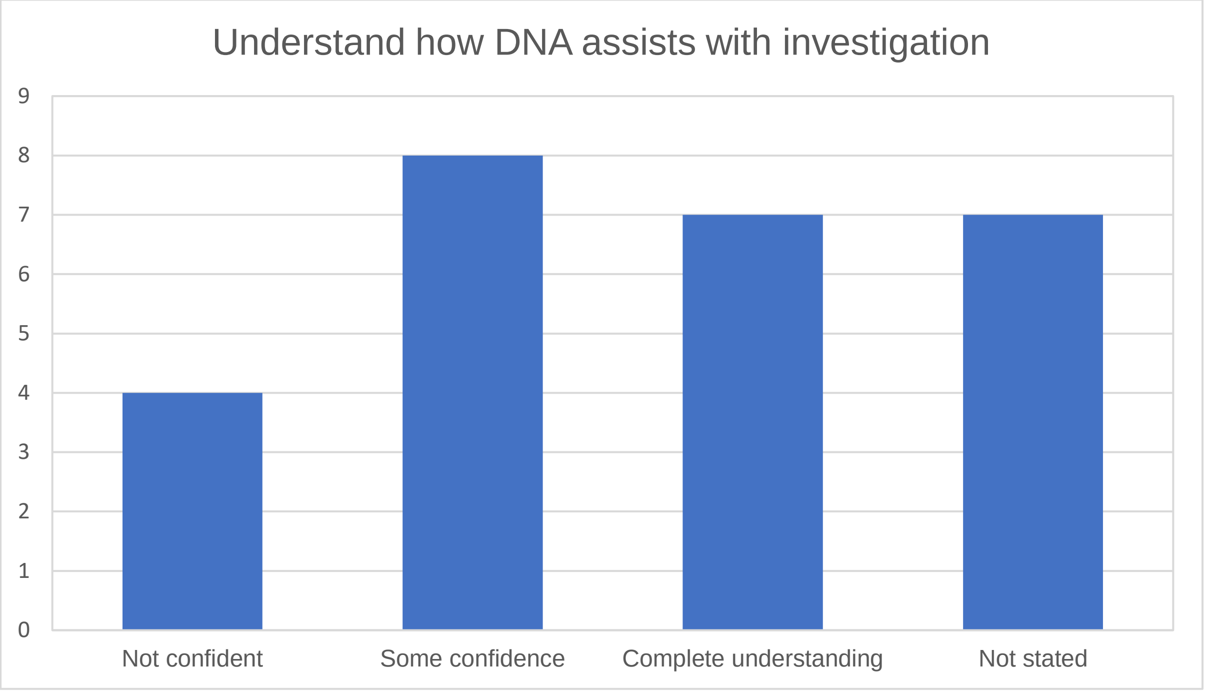

Supplement: Supplementary file 1 [file Data_Sheet_1.pdf]
